# Isolated Fetal Ventriculomegaly: Diagnosis and Treatment in the Prenatal Period

**DOI:** 10.3390/children11080957

**Published:** 2024-08-08

**Authors:** Mateusz Zamłyński, Olena Zhemela, Anita Olejek

**Affiliations:** 1 Department of Gynecology, Obstetrics and Oncological Gynecology, Faculty of Medical Sciences in Zabrze, Medical University of Silesia, Stefana Batorego 15, 41-902 Bytom, Poland; anitaolejek@wp.pl; 2 Department of Obstetrics and Gynecology, Danylo Halytsky Lviv National Medical University, 79010 Lviv, Ukraine; olenaz2008@ukr.net

**Keywords:** fetal ventriculomegaly, prenatal surgery, isolated ventriculomegaly, cephalocentesis, ventriculostomy, severe ventriculomegaly

## Abstract

Fetal ventriculomegaly (VM) is a defect of the central nervous system, typically diagnosed during the second-trimester ultrasound in fetuses with an atrial diameter (AD) of >10 mm. Non-isolated ventriculomegaly (NIVM) is heterogeneous in nature, coexisting with additional intracranial and/or extracranial malformations and genetic syndromes, resulting in an unfavorable prognosis for the further development of the child. Both the pregnancy management and counseling are dependent on the findings of combined ultrasound/MRI, genetic testing, and gestational age at diagnosis. The purpose of this review is to propose a hypothesis that diagnostic advancements allow to define the process of identification of the isolated forms of VM (IVM). Based on the evidence presented in the literature, we consider whether prenatal decompression for severe isolated VM (ISVM) is supported by the experimental trials and whether it might be implemented in clinical practice. Also, we describe the evolution of the diagnostic methods and expert opinions about the previously used prenatal decompression techniques for ISVM. In conclusion, we introduce the idea that fetal surgery centers have either reached or nearly reached the necessary level of expertise to perform such procedures. Endoscopic cystoventriculostomy (ETV) appears to be the most promising, as it is associated with minimal perinatal complications and favorable neurological outcomes in the neonatal period. Randomized trials with long-term neurodevelopmental follow-up of children who underwent prenatal decompression due to ISVM are necessary.

## 1. Introduction

Fetal ventriculomegaly (VM) is a defect of the central nervous system (CNS), characterized by an excessive accumulation of cerebrospinal fluid (CSF) in the brain ventricles. VM is caused by an absent or inadequate CSF flow, from its point of production in the ventricles to the site of absorption into the systemic blood circulation. VM is merely a manifestation of a medical condition, not a diagnosis, as the etiology of the disease remains heterogeneous, with a broad spectrum of unfavorable neurodevelopmental outcomes [1]. The causes of congenital VM include a narrowing of the aqueduct of Sylvius, known as aqueductal stenosis (AS), porencephalic cysts, myelomeningocele, Dandy–Walker malformation, as well as atresia of the foramina of Magendie and Luschka [2,3]. The prevalence of VM diagnosed prenatally has been estimated at 0.3–1.0/1000 live births [4]. Other causes of hydrocephalus, including cerebral and extracerebral, need to be excluded in order for the diagnosis of isolated fetal ventriculomegaly (IFVM) to be made [1]. Prenatal diagnosis of IFVM is preliminary and merely ‘probable’, as further postnatal diagnostics may in time reveal genetic disorders of the central nervous system (CNS) [2]. Therefore, the prenatal diagnostic pathway is a time- and resource-consuming, complex procedure that may only determine the ‘apparent’ type of IVM [4]. Due to the lack of prenatal decompression paradigms in the diagnosis of ISVM, current IVM pregnancy counseling is limited to the option of continuing the pregnancy with the prospect of further postnatal treatment, or TOP [1]. The findings of studies about agent-induced VM and prenatal decompression in animal models as well as clinical series of decompression using various surgical techniques are the reasons why a ‘second look’ at the validity of continuing with the 30-year IFSMA moratorium seems prudent. The aim of this review is to present the new diagnostic methods of identifying IVM and the advancements made in ISVM decompression techniques, which may be applicable in the prenatal period.

A randomized, MOMS-like trial in the future might provide evidence to support a change in the current clinical practice.

## 2. Diagnostic Pathway for Fetal IVM

### 2.1. Key Findings of the Width of the Lateral Ventricles of the Fetal Brain

VM is classified into ‘mild’ (10–12 mm), ‘moderate’ (13–15 mm), or ‘severe’ (>15 mm) forms [5,6,7] on the basis of AD measurements. In order to avoid confusion and achieve more clarity during parent counseling, the division into ‘mild’ (AD 10–15 mm) or ‘severe’ (AD > 15 mm) forms is also used due to similar neurodevelopmental results [8,9,10]. If no additional abnormalities are detected, these types are defined as ‘isolated non-severe VM’ (INSVM) and ‘isolated severe VM’ (ISVM), respectively [11,12,13].

### 2.2. Ultrasound Neuroscan for Fetal VM

The diameter of the lateral ventricles at the level of the atrium is commonly referred to as the atrial diameter (AD)—the standardized width of where the frontal and temporal horns of the lateral ventricles converge [14,15].

On the second-trimester neuroscan, AD of >15 mm, dilated third ventricle in transverse dimension (>2 mm), the so-called ‘dangling’ choroid plexus, normal posterior cranial fossa and fourth ventricle, and normal or enlarged cranial biometry are the hallmarks of severe VM [16,17,18]. A recent three-dimensional transvaginal neurosonography study demonstrated a strong correlation between the diameter of the third ventricular interatrial fusion of <7.1 and a postnatal diagnosis of AS (98.6% CI, 0.92–0.99) [19].

Fetuses with moderate VM at the beginning of the second trimester should undergo repeated neurosonographic examinations, as the risk of ISVM progression to SVM is observed in 13–16% of the cases [20,21,22].

### 2.3. MRI in Fetal VM Diagnosis

Magnetic resonance imaging (MRI) is usually a second-line test, complementary to the neurosonography performed in the second trimester of pregnancy [23,24,25]. The T2-dependent contrast technique using fast (turbo) spin–echo (SE) or steady-state free-precession (SSFP) sequences is the primary method for prenatal CNS imaging. Axial diffusion-weighted imaging (DWI), used to study the cellularity of the brain tissue and water movement, which is helpful in the evaluation of brain infarction or hemorrhage, is an example of the latest MRI [26]. Also, echo planar imaging (EPI) allows to achieve higher diagnostic sensitivity in intracranial hemorrhage [27].

Fetal MRI provides excellent soft tissue resolution on spatial imaging. Additional anomalies diagnosed by MRI mainly include dysgenesis of the corpus callosum, heterotopia, and abnormal cortical folding [28,29].

MRI provides key evidence in distinguishing between isolated and non-isolated VM, both for counseling and the eligibility process for ventriculo-amniotic shunting (VAS). Heaphy-Henault et al., in their MRI study of 43 fetuses, reported specific features of AS causing isolated VM, with funnel-shaped aqueduct morphology, hemorrhage within the aqueduct, dilatation of the inferior recesses of the third ventricle, as well as abnormal thinning and dysgenesis of the corpus callosum [30]. Increased intracranial pressure results in a thinning of the cortical parenchyma, with the development of a thin cortical mantle and loss of intracranial extra-axial spaces [31]. In a recent multicenter MRI study of 187 fetuses with the prenatal diagnosis of ISVM, additional structural abnormalities were detected on neurosonography in the cortex in 32.4% of the cohort, whereas midline or acquired lesions (hypoxia or hemorrhage) were detected in 26.5% and 14.7% of the cases, respectively [32].

### 2.4. Compatibility of Neurosonography and MRI

Ultrasound, being a non-invasive, inexpensive, easily reproducible, and time-efficient technique, is a widely available imaging modality [16,18]. Fetal MRI offers the added value of detecting additional CNS malformations, with a detection rate of 6.2–14.5% [31,32,33]. The most common types of anomalies include cortical malformations and midline abnormalities [32,34,35].

**Figure 1:**
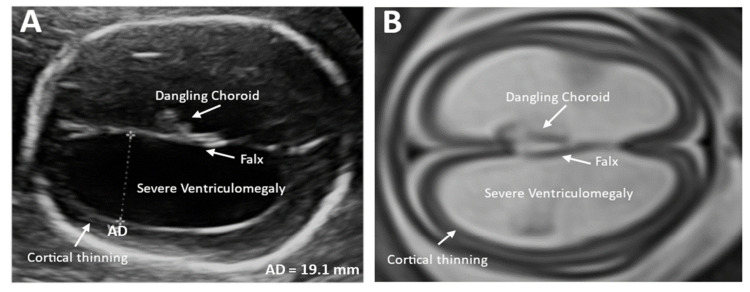
Imaging in the transventricular plane of ISVM at 22.3 weeks of pregnancy (**A**) transabdominal ultrasound: the atrial width of the distal ventricle is increased AD = 19.1 mm, (**B**) MRI T2-dependent sequence SSFSE.

A new combination of 3D-US and MRI-3D-Slicer techniques is used to assess the spatial volumetry of the four ventricular horns and determine the course of developmental changes in the fetal brain. Knowledge about the developmental evolution of the shape and size of the ventricular horns is essential in the diagnostic process of isolated VM because it can improve prenatal counseling, management strategies, and pregnancy outcome [36]. In addition, MRI detects IAS comorbidities such as fetal cerebellar malformations (fCM), ACC, cerebellar or cortical mantle anomalies, and absence of ventricular septum [18,37]. According to several sources, in rare cases of severe IVM (2/10,000 births), the reported incidence of additional structural abnormalities detected by fetal MRI alone ranges from 18.1% to 57% [10,38,39]. In summary, the use of MRI in the prenatal diagnostic protocol for fetuses with isolated VM seems to facilitate more accurate assessment of ventricular width and also allows to detect other brain abnormalities, which might significantly affect the course of further clinical management. An illustrative comparison of US and MRI of ISVM is shown in Figure 1.

## 3. Methods of Diagnosing Genetic Disorders in VM

Genetic testing is fundamental in the process of diagnosis and counseling, which establishes prenatal treatment and postnatal prognosis [40]. The exclusion of an abnormal fetal genotype is the only factor that supports the diagnosis of IVM [41]. Genetic testing of chorionic villus biopsy material or the amniotic fluid cells of fVM should also include genotyping with chromosome microarray analysis (aCGH) of the number of DNA copies for defects associated with primary aneuploidies 13, 18, 21 XY, with the possibility of detecting microdeletions and microduplications in 6.7% of the cases [42,43,44].

According to Toren et al., CGH microarray aberrations occur in non-isolated VM with a prevalence of 24.1% as compared to 6.2% in ISVM (*p* = 0.031). The prevalence rate of genetic aberrations is not related to the degree of ventricular dilatation [45].

The technique of exome sequencing (ES), whole exome sequencing (WES), or whole genome sequencing (WGS) is the final frontier in genetic studies of CNS defects [46,47,48]. In the Population Architecture through Genomics and Environment (PAGE) study of 610 fetuses with structural anomalies, the WES test identified a genetic variant in only 8.5% of the fetuses with an isolated brain anomaly, which was lower than expected based on the previous studies with smaller cohorts [49]. Monogenic mutations found in ACC are usually associated with neurodevelopmental disorders secondary to the mutations in the EPG5, ZEB2, SLC12A6, and AIC genes [35,50,51].

The most common hereditary form is the X-linked hydrocephalus with the stenosis of the aqueduct of Sylvius, known as the L1 syndrome, caused by changes in the L1CAM gene [52,53]. L1CAM mutations are observed in aqueductal stenosis. Other mutations associated with congenital VM include MPDZI encoding MUPP-1, a tight junction protein, and CCDC88C mutations encoding DAPLE in the Wnt signaling pathway [54]. Ultimately, ES, WES, and WGS testing provides important support for counseling offered at centers with adequate resources and experience [46,50,51]. These tests should be dedicated to patients with a familial history of malformations, especially when considering prenatal ISVM drainage options. However, the abovementioned studies do not conclusively determine the relationship between exome and phenotype [46].

## 4. Infectious Factors in the Development of Fetal VM

The prevalence of intrauterine infection as the causative factor for fetal VM has been estimated at 1.4% [55]. Cytomegalovirus and toxoplasmosis remain the most common infectious causes concomitant with VM [56]. Viral infections such as rubella, parvovirus B19, Zika, and herpes simplex can cause VM, so testing should be obligatory for women from high-risk groups [57]. A positive test result for TORCH IgG and IgM, if concomitant with VM or other developmental anomalies, is an indication for polymerase chain reaction (PCR) testing of the amniotic fluid [58].

## 5. Rationale behind Prenatal Ventriculo-Amniotic Shunting in ISVM

The current neurosurgical treatment of choice for congenital obstructive ISVM includes postnatal CSF drainage via ventriculoperitoneal shunting (VPS) or an endoscopic third ventriculostomy (ETV) to bypass the obstruction and redirect the CSF flow [11,59,60]. Alas, postnatal decompression of the brain tissue is a delayed intervention because the critical period of brain development had already taken place prenatally, while the neuronal migration and molecular consequences became irreversible, impairing motor and neurocognitive outcomes in the affected patients [61]. ISVM causes an increase in the intracranial and/or intraventricular pressure, disrupting the hemodynamics and the metabolism of the brain tissue. Glick et al. demonstrated that increased CFS pressure is responsible for decreased cerebral blood flow, regional ischemia, and metabolic changes in the periventricular neurons [62]. Obeidat et al. performed a Doppler examination of the middle cerebral artery and found abnormal absent or reversed diastole flows in fetuses with VM. Additionally, these authors showed a significant correlation between cerebral circulatory abnormalities due to increased intracranial pressure and high perinatal mortality in those fetuses [63].

Also, postnatal implantation of the drainage systems is associated with a high incidence of shunt failure: approximately 30% in the first year and 10% per year in the subsequent years [64]. Intracranial pressure cannot be assessed during prenatal life. Therefore, when the enlargement of the ventricular size meets the criteria for SVM, one may suspect an obstructive type of VM with elevated intracranial pressure, which cannot be diagnosed prenatally [65]. In fact, only 10–15% of the newborns present with signs of elevated intracranial pressure postnatally [66]. A similar opinion was reported by the ENSO Working Group, which confirmed that the neurological outcomes are strongly correlated with the severity of VM, with the overall survival rates decreasing from 93 to 96% in mild to 28% in severe VM [18].

The devastating effects of SVM on the brain tissue have been well recognized by histopathological studies. Pathological changes include loss of parenchyma and embryonic matrix, with destruction and glial changes in the periventricular axons and secondary loss of neurons, axonal degeneration with focal loss of neurons, and a decrease in synaptic density [67]. Abnormal myelination may result from ischemia and/or the toxins present in the cerebrospinal fluid [68].

AD progression in IVM is highly individual and requires repeated ultrasound measurements. Rault et al. described three patterns of prenatal sonographic appearance of AS: rapid progression in the third trimester, progressive VM in the second and third trimesters, and stable VM [69]. Perlman et al. compared the results of prenatal and postnatal imaging in a cohort of 92 fetuses with mild-to-moderate IVM (AD ≥ 10 and <14 mm) and found a statistically significant reduction in the ventricular width (*p* < 0.001). The patients were followed up for 24 months, and during that time all children showed normal development, with the exception of three with very mild neurological deficits [70].

In contrast, prenatally, fetuses with ISVM present with AD of ≥15 mm, which found at any period of gestation may indicate stabilization, progression, or even reduction in the AD values [65]. The dynamics of fetal ISVM development do not have a uniform pattern. Kline-Fath et al., in their MRI study of 30 fetuses with AS, found that progressive SVM resulted in ventricular rupture (60%), loss of septum pellucidum leaflets (47%), and reduction in the white matter and corpus callosum volume (43%). Cerebellar ectopia developed in 27% of the fetuses, and 6% met the criteria for Chiari I malformation [71]. Postnatally, 97% of the neonates required VP shunting. The authors determined that the rate of AD progression from the time of the prenatal SVM diagnosis to birth was 1.2 mm/week [71]. Ge et al., in a small cohort of 36 ISVM fetuses, used serial neurosonography to determine the weekly rate of AD progression for two groups: ventricular acceleration of ≥3 mm/week vs. plateau of <3 mm/week. The comparison of mean AD progression between ≥3 mm/week vs. <3 mm/week was 4.1 mm/week vs. 1.0 mm/week, respectively (*p* = 0.031) [72]. All patients with the increase of ≥3 mm/week required VPS [72]. These findings provide information for identifying cases that might benefit from prenatal shunting.

## 6. CNS Damage Caused by Fetal VM

The cumulative result of neurological deficits caused by CNS malformations is the effect of the mother–placenta–fetus triad in early pregnancy [73]. Developmental neuroplasticity of the brain tissue is more likely to occur during the critically sensitive periods of brain maturation within the first 1000 days after conception [73]. The First Thousand Days fetal–neonatal research program, by using the 1000-day observational perspective, identifies pregnancy-specific mechanisms affecting the mother–placenta–fetus triad, expressed as brain malformations and destructive changes from the second and third trimesters throughout the second year of life [74].

According to the SMFM Consult Series guideline, cortical destruction in mild or moderate VM is associated with a low risk for neurosurgical postnatal intervention and favorable neurodevelopmental outcomes in >90% of mild and 75–93% of moderate VM cases [12]. However, even in confirmed isolated mild-to-moderate VM, developmental malformations of both gray and white matter have been described [75]. MRI of 41 fetuses with INSVM (AD 10.0–12.0 mm) revealed abnormal development of the transient fetal brain zones belonging to both hemispheres, regardless of the VM-affected side [75]. In further studies, it seems justified to use the uniform term of INSVM, with an AD of 10.0–14.9 mm, to evaluate fetal and neurodevelopmental outcomes as it provides more clarity. Benkarim et al., in their MRI study of 23 INSVM fetuses, described cortical gyrification, which remained dependent on the ipsilateral position of the ventricular enlargement associated with reduced cortical folding [76]. Hahner et al., in their MRI study of 32 fetuses with INSVM, assessed global and regional changes in the cortical development and found differences in the development of the gray matter as well as decreased cortical volume in the frontal lobes associated with neonatal neurobehavior [77]. The abovementioned authors concluded that changes in the cytoarchitecture may affect neuronal interaction and functionality, which later in life might be associated with schizophrenia, ADHD, and attention deficit disorders [76,77,78]. In a new study by Kyriakopoulou et al., in a cohort of children prenatally diagnosed with VM, 9 out of 24 (37.5%) met the criteria for autism spectrum disorder (ASD). A statistically significant correlation was detected between VM and the ADOS-2 autism/ASD classification (*p* = 0.024). A neurodevelopmental study in children with fetal IVM with impaired cortical development reported the following ASD traits: difficulty to sustain attention, working memory, and cognitive behavior [79].

The results of the studies with MRI during the perinatal period in fetal INSVM help to identify groups of children and adults who require further long-term follow-up [35].

## 7. Evaluation of Biomaterials and SVM Decompression Techniques during the ‘Post-Moratorium Era’

### 7.1. Serial Cephalocentesis and VAS

In the previous century, the initial attempts at prenatal decompression of SVM using craniotomy were intended to reduce the circumference of the fetal head, thus enabling vaginal delivery [80]. Similarly, repeated US-guided percutaneous aspirations of the CSF to reduce the risk of intrapartum maternal morbidity and mortality, with no therapeutic effect on the fetus or the neonate, were also reported [81]. The development of the US-guided neuroscanning technique in the 1970s and the possibility of repeated observations indicating a progressive fetal cerebral degeneration in SVM led to the idea of permanent prenatal ventricular shunting [82]. Harrison et al. introduced into clinical practice the concept of a single implantation of a unidirectional VAS system or an expanded pressure-valve system into the fetal brain [83]. In 1982, Clewell et al. described the placement of a silastic shunt with a unidirectional valve in a patient with fetal VM using the US-guided percutaneous needle [84]. Early attempts to perform percutaneous US-guided VA shunting resulted in 10% fetal mortality during the procedure and moderate-to-severe disability in 66% of the survivors [85]. In 1982, the International Society for Fetal Medicine and Surgery established a voluntary International Fetal Surgery Registry [86]. Between 1982 and 1985, 39 cases of in utero shunting were reported to the registry, with an overall survival rate of 83% and procedure-related mortality of 17%. More than half of the patients were presented with severe neurological deficits; 12% had mild-to-moderate impairment, and only 35% had no developmental delays [68]. In 1986, higher mortality rates among patients who underwent shunting in utero as compared to postnatally, as well as poor neurodevelopmental outcomes of the perinatal shunting survivors, prompted the members of the IFSSM community to establish a voluntary ‘moratorium’ against VAS until evidence of shunting efficacy became available [85,86].

A series of experimental trials in animal models with induced AS to determine the feasibility of neuroendoscopic (EVT) decompression through the Monro orifice to achieve fenestration of the third ventricle heralded significant advances in prenatal VM management [87]. Peiro et al. injected polymeric agents (E85, E105) into the third ventricle and induced hydrocephalus in 50 lambs [87]. The point of entry for the endoscope was anterior to the coronary suture, 7 mm from the midline; localization at the bottom of the third ventricle was achieved in 32 out of the 50 cases (64%). In 36% of models, polymers in the CSF or bleeding from the choroid plexus were reported. Total prenatal perforation of the third ventricle using the ETV method was achieved in 80% (32/40) of the models [87].

Advances in biomaterial engineering are directed at creating a non-immunogenic shunt that, after ultrasound-guided prenatal implantation, will not be prone to obstruction, migration, and infection [88]. The previously used Orbis-Sigma and Accu-Flow shunt systems and Cook fistulas meet the conditions of the unidirectional CSF flow and are equipped with a system that prevents overdrainage. Alas, due to shunt migrations, they required repeated implantations, which may be associated with a high risk for PTL (preterm labor) [85,89].

In 2016, Chen et al. presented a ventriculo-amniotic shunting device for treating fetal aqueductal stenosis, constructed out of 3Fr or 4Fr catheters that had longitudinal bending stiffness, resistance, sufficient lumen area for CSF drainage, and capacity for valve integration [90]. The most recent experimental improvement to VAS was a full valve system where the valve contains a silicone–nitinol composite main tube, a super-elastic anchor with a 90° angled dual dumbbell, and an ePTFE valve encased by a stainless steel cage. The anchor changes its diameter from 1.15 mm (coiled state) to 2.75 mm (uncoiled state), demonstrating an up to 1.4-fold diameter change in human body temperature, which prevents shunt migration [91]. The functionality of these devices, resulting in the reduction in high intracranial pressure and the prevention of device migration and CSF reflux, has so far reached the pre-clinical trial phase [90,91].

The voluntary IFMSS ‘moratorium’ against prenatal fetal ventricular decompression in SVM, which has been in force for thirty years, did not result in complete abandonment of the procedure [11]. In countries where termination of pregnancy (TOP) is prohibited by law, such as Brazil and Poland, VAS implantation has been performed with the approval of the local bioethics committees [89,92,93]. Cavalheiro et al. described 39 fetuses between 24 and 32 weeks of gestation, diagnosed with SVM, and treated with intrauterine therapy [92]. VAS implantation was performed in 19 fetuses using the KCH-Rocket pigtail system. After birth, almost all patients (38/39) required VP shunting with low-pressure valves. These authors reported a total of 57 SVM decompressions performed with the use of the following techniques: serial cephalocentesis, VAS implantation, and one successful EVT procedure (Table 1) [93]. Neurodevelopmental assessment at year three of life revealed normal development in 66%, moderate impairment in 15%, and severe delay in 19% of the followed-up cases [93].

The Polish fetal therapy group from the ICZMP (Polish Mother’s Memorial Hospital Research Institute) in Lodz has performed VAS implantations in 222 fetuses with SVM since 1992 [91]. The learning curve for that center for 2006–2012 shows a 50% reduction in the AD values, with a subsequent increase in the cortical width reported for 25% of the cases. Complications were found in 22 VAS patients, including four intrauterine deaths at a mean GA of 34 weeks at birth. Nearly 18% of the newborns did not require any additional neurosurgery. Favorable neurological outcomes were reported for 60% of the infants [90]. The same center reported the results of 44 VAS procedures performed at a mean GA of 25 weeks, with the inclusion criterion of AD >20 mm and confirmed isolated SVM [94]. These authors reported the following complications: three deaths after VAS implantation; in 50% of the cases, the implant (Reusable Introducer Set; Rocket Medical plc, Washington, UK) migrated into the amniotic or ventricular cavity. Still, the authors also reported 41 live births at a mean GA of 37 weeks. In addition, neurodevelopmental assessment of the survivors with ISVM at age 2 on the Bayley scale revealed that 19 (70.4%; CI95%, 51.5–84.2%) children had a normal or mild neurodevelopmental delay, and 8 (29.6%; CI95% 15.6–48.5%) were moderately or severely delayed. In contrast, out of 11 children with NISVM, 2 (18.2%; 95% CI 5.1–4.8%) had a normal or mild neurodevelopmental delay, and 9 (81.8%; 95% CI 52.3–94.9%) were moderately or severely delayed [94].

**Table 1:** Chronological list of reports about prenatal decompression techniques used during the ‘post-IFMSS moratorium era’.

Author	Perinatal Center	Year	Total Number of Patients Undergoing Surgery	Cephalocentesis	Percutaneous VAS/USG	OFS	EVT
Cavalheiro [92]	Sao Paulo, Brasil	2003	39	20	19	-	-
Bruner [95]	Nashville, TN, US	2006	4	-	-	4	-
Al-Anazi [96]	Al-Kobar, Saudi A.	2007	1	-	-	1	-
Al-Anazi [97]	Al-Kobar, Saudi A.	2010	5	-	-	5	-
Cavalheiro [9]	Sao Paulo, Brasil	2011	57	26	30	-	1/3 attempts
Szaflik [89]	Łódź, Poland	2014	222	-	222	-	-
Litwińska [94]	Łódź, Poland	2019	44	-	44	-	-
Peralta [98]	Sao Paulo, Brasil	2023	10	-	-	-	10
Zamłyński [99]	Bytom, Poland	2024	1	-	-	1	-

### 7.2. Endoscopic Third Ventriculostomy ETV

In 2003, Cavalheiro et al. reported the first case of endoscopic third ventriculostomy performed in a fetus using a percutaneous technique under US guidance [92]. A 2.5 mm trocar was inserted into the lateral ventricle through the anterior fontanelle, as well as a 2.3 mm neuroendoscope with a 1 mm working channel (Neuroview, flexible scope, 25C, Traatek, Fort Lauderdale, FL, USA). A Fogarty 2Fr catheter was introduced into the third ventricle through the foramen of Monro and the Liliequist membrane, achieving CSF drainage. The cesarean section was performed at 38 weeks, and the fetus was successfully delivered [92]. In 2023, Peralta et al. described 10 EVTs in human fetuses using the percutaneous technique [98]. Fetuses with progressive apparent ISVM at median GA of 28.7 weeks (25.3–30.7) were found eligible for surgery. These authors reported that 70% (7/10) of the fetuses had reduced or stabilized lateral ventricular atria. Median GA at delivery was 38.2 weeks (35.9–39.3). EVT was performed using a 2.4 mm two-channel straight sheath with a sharp tip (11530 KA, Karl Storz, Tuttlingen, Germany), a 1.2 mm semi-rigid endoscope (11540 AA, Karl Storz, Germany), and a 2F-Fogarty balloon catheter (Edwards Lifesciences, Mississauga, ON, Canada). The sheath was advanced through the lateral apex of the bregmatic fontanelle to reach the anterior horn of the lateral ventricle. The ventricular pressure of the CSF and the amniotic fluid were measured. A Fogarty catheter perforated the base of the third ventricle, and the balloon was inflated with 0.2 mL of saline to create a communicating orifice. Postnatally, 80% of the children required VP shunting. Children whose AD stabilized after prenatal ETV presented with better neurodevelopmental outcomes as compared to the group with higher scores on the ASQ-3 test: 57.1% (4/7) vs. 100% (3/3), respectively. Further studies are necessary to determine whether earlier intervention at 27 weeks of GA in fetuses with lower AD ISVM values might improve the developmental prognosis of these children. The results of the ETV experimental studies reported by Peiro et al. and Peralta et al. proved the feasibility of using this minimally invasive procedure in the treatment of ISVM in human fetuses. Their findings represent a milestone in neurosurgical research. However, the neurological development of the children who had undergone prenatal ETV should be confirmed in long-term follow-up.

### 7.3. Applications of Open Fetal Surgery Techniques for SVM Decompression

In an attempt to summarize the outcomes of the prenatal SVM decompression techniques applied so far, one should mention several teams of innovators in the field of SVM decompression. As early as 1986, Michejda et al. presented an innovative—at the time of publication—hypothesis, claiming that “[…] the inadequacy of current shunting methods in hydrocephalus suggests that an open technique via hysterotomy may be a more effective method of placing a suitable ventriculoperitoneal shunt and may in fact become the treatment of choice for well-selected progressive fetal hydrocephalus” [100]. A total of 11 open fetal surgeries for the implantation of the valve systems have been performed by their team until today (Table 1).

Al-Anazi et al. described six cases of the so-called ‘Al-Anazi VA shunt’ implantation, using a unidirectional valve of 3 cm, equipped with wings to prevent migration of the system, inserted through a 1 cm hysterotomy [96]. The first implantation was performed at 32 weeks of GA and resulted in an overdrainage of the ventricles, which required placement of a VP shunt after birth in an infant delivered at 37 weeks of GA [97]. In the remaining five pregnancies, implantation was performed at 27–31 weeks of GA. All shunts effectively drained the CSF into the amniotic cavity along the pressure gradient. Further clinical trials were eventually abandoned due to the reported complications: placental ablation, shunt infection, sepsis, and severe developmental delays [95,97].

Between 1999 and 2003, Bruner et al. performed shunt implantation via a hysterotomy between 23.0 and 26.5 weeks of GA in four fetuses with obstructive AS at the Vanderbilt University Medical Center, with the approval of the Institutional Review Board [95]. The eligibility process included MRI, serial measurements of the AD values using ultrasound, which revealed an average growth of 11.3 mm/week, genetic testing (karyotype and aCGH), microbiological examination of the amniotic fluid, consultation with a genetic counselor, and a prospective evaluation of the further development of the child. The uterus was exteriorized using the Pfannenstiel incision. A complex drainage system: ventricular catheter, low-pressure valve, PS Medical Ultrasmall^®^ (Medtronic, Dublin, Ireland), was inserted with a needle using the Seldinger technique after making a 1 cm scalp incision above the right ear at Keen’s point. The distal part of the catheter was tunneled subcutaneously to the amniotic outlet in the fetal neck area, with the possibility of repositioning to VAS after the delivery. The deliveries took place between 27 and 34 weeks of GA. All shunts displayed good draining properties until delivery, validating the feasibility of shunt placement and maintenance. Postnatally, three patients underwent drain repositioning to VPS. Infection was the main reported complication, affecting three out of the four patients, as well as one death during the neonatal period. Unfortunately, all patients presented with neurodevelopmental delay [95].

**Figure 2:**
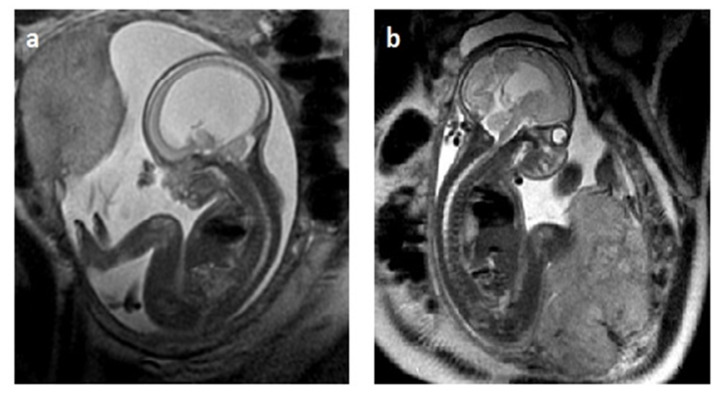
A comparison of the fetal MRI in the sagittal plane, depicting the change in the lateral ventricular size of the fetal brain (**a**) pre-VAVI vs. (**b**) post-VAVI [99].

In a recent publication from our center, Zamlynski et al. described intrauterine ventricular–amniotic valve implantation (VAVI) with OFS, performed at 24.4 weeks of GA [99]. In the eligibility process for OFS, the following MOMS (Management of Myelomeningocele Study) exclusion criteria were used for the mothers: cervical insufficiency and/or short cervix (<20 mm on US), history of PTL (delivery at <37 GA), congenital anomalies of the Muller’s duct, postoperative uterine deformities, type 1 diabetes mellitus, maternal–fetal Rh(D) isoimmunization, obesity defined as BMI ≥ 35 kg/m^2^, anti-HIV or anti-HCV seropositivity, HBV viremia, other serious maternal illnesses, psychosocial problems, lack of a support system, and/or inability to travel and participate in the follow-up [101]. Fetal eligibility criteria included ISVM diagnosed on MRI—supratentorial VM with no signs of CSF flow through the cerebral ventricles, absence of abnormal karyotyping of the amniotic fluid cells on aCGH, or other than IFV anatomical abnormalities. Repeated US measurements showed progressive VM: 2 mm/week, AD from 18.3 to 22.6 mm. The abdominal cavity was opened using the Pfannenstiel incision without exteriorizing the uterus. VAVI was implanted using the technique described by Bruner et al. [95]. The 46220 Shunt Ultra Small 4CM LL (Medtronic PS Medical, Goleta, CA, USA) kit was used. ISVM decompression to AD of 13.6 mm and a favorable change in parenchymal thickness of 4.3 mm vs. 8.0 mm were achieved. After delivery at 31.1 weeks of GA due to the pPROM syndrome, the valve required evacuation due to signs of infection. Next, SVM drainage was achieved by insertion of the Rickham reservoir, with conversion to VPS. At age 7, the child presented with mild-to-moderate developmental delay and atypical autism. WGS revealed a variant in the ZEB2 gene (NM_014795.4(ZEB2):c.2813C>G. [99]. A comparison of the fetal MRI pre- and post-VAVI is shown in Figure 2.

### 7.4. FSC Criteria for VA Shunting

Potential ISVM recipients of the prenatal implantation of a valve system should be diagnosed and hospitalized at referral centers that offer the highest available level of care. The criteria for the highest-level credentials were presented in a recent publication, ‘Care Levels for Fetal Therapy Centers’ by Baschat et al., in the required resources category for fetoscopic interventions and surgery with unavoidable maternal laparotomy [102]. Similarly, Moldenhauer et al. highlighted the necessity of applying for accreditation with well-documented evidence of a positive learning curve by outlining the requirements for the highest level of care fetal surgery center [103]. However, the catalog of fetal prenatal interventions listed in the literature does not include VAS implantation techniques, which might be applied in ISVM therapy.

### 7.5. Model Candidates for Prenatal VA Shunting

Maternal–fetal surgery (MFS) is a specific, advanced procedure that affects the mother as well as the fetus. The probability of maternal complications must be analyzed by an internal medicine specialist and an anesthesiologist. All maternal concerns regarding her condition and the validity of the procedure should be addressed, with the help of psychological and/or ecumenical support, if needed [1]. The MOMS protocol exclusion criteria should be applied to all mothers who are candidates for MFS for ISVM shunt implantation via hysterotomy [101]. Surgical and perinatal complications should be reported using the standardized Clavien–Dindo and WHO scales [104,105].

It is the fetus with ISVM who is the actual MFS patient and the candidate for the VAS procedure. A series of advanced neuroscans is required to demonstrate the dynamics of AD growth, at the rate of 2 mm/week to >20 mm/week, with degenerative thinning of the cortical mantle [70,71,72,73]. Concomitant CNS and extra CNS defects need to be excluded using combined MRI and US imaging. Inconclusive findings of the infection tests need to be clarified by PCR testing of the amniotic fluid samples. Genetic testing should include fetal amniotic fluid cells with aCGH genotyping. Advanced WES or WGS testing may be considered in patients with suspected additional defects or complicated familial medical history [46,52,54]. As far as counseling for the parents of an ISVM child is concerned, it is vital to mention that 37% of the affected children survive without disabilities [106]. Last but not least, the parents should also be informed about the risk for additional developmental diagnoses later in life, even in cases with ‘the most apparent’ ISVM.

### 7.6. Optimal Timing of Prenatal ISVM Shunting

Knowledge about the natural history of fetal VM and the results of experimental trials on animal models helped to define two clinical and pathomorphological stages of the disease, allowing to determine the optimal timing for shunting in patients with ISVM. The first stage of fetal VM is characterized by supratentorial rupture and swelling of the periventricular white matter and of the axons. During the second stage, irreversible gliosis and demyelination are observed [107]. If this evolution is stopped by ventricular fistula in the initial period or rupture of the lining and the ependyma of the white matter, complete reconstitution of the brain tissue may occur, with excellent results. Nevertheless, the results may be disappointing if the same treatment is applied during the second stage, when gliosis and demyelination are already present [108]. Also, treatment of kaolin-induced VM in animal models by inserting a shunt or a VAS valve via hysterotomy increased the rates of normal development and survival as compared to expectant management [107,108,109]. There is a well-established consensus about the need for the earliest possible treatment, as it benefits the motor and cognitive development of the fetus [107,108,109]. In terms of eligibility and gestational age, fetuses with progressive ventricular enlargement and cortical thinning before 28 weeks GA may be better candidates because after 32 weeks the brain damage is most likely irreversible [110].

### 7.7. Benefits and Limitations of ISVM Shunting Techniques

Due to the lack of well-established prenatal treatment paradigms and patient selection criteria, the 30-year IFMSS ‘moratorium’ resulted in a decline in the number of shunt placements [111]. Still, significant progress in MFS was achieved during that time. These advancements were the result of numerous improvements in fetal imaging techniques, the development of fetoscopic instrumentation, material bioengineering, and, above all, reduced invasiveness of the procedures [81,89]. Experimental studies confirmed the effectiveness of prenatal EVT in draining ISVM and reported 11 cases of surgery in human fetuses [9,98]. In some patients, EVT in infancy is allowed to avoid long-term shunting [112,113]. The chronological list of the reported prenatal decompression techniques during the ‘post-IFMSS moratorium era’ is shown in Table 1.

## 8. Conclusions

### 8.1. Current State of Knowledge about Fetal IFVM

Increased accessibility of ultrasound technology has facilitated earlier diagnosis of fetal ventriculomegaly (VM), with detection possible as early as the first trimester of pregnancy. Dedicated repeated neurosonography and advanced MRI may identify groups of patients presenting with non-severe (AD 10.0–14.9 mm) and severe (AD ≥ 15 mm) forms of VM. Isolated VM can only be diagnosed after the exclusion of additional cranial and extracranial malformations and by using genetic testing from chorionic villus sampling, amniotic fluid karyotyping, and aCGH microarray. Although ES—WES and WGS—sequencing provides a significant body of evidence, the correlation between prenatal genotype and postnatal phenotype requires further studies. Active infectious agents of the TORCH group should be excluded.

During counseling for INSVM, it is vital to mention the chances for a favorable outcome, with low risk for neurosurgical interventions later in life, and the obligatory long-term CNS follow-up using standardized neurological tests.

ISVM with AD progression and fetal HC of >95th pctl. is associated with high perinatal mortality and poor neurodevelopmental prognosis.

Counseling for all patients with VM should put emphasis on the transient nature of even the most apparent isolated types of VM. The parents should be informed about the high probability of additional medical diagnoses, including ADHD and autism spectrum disorders, for their child. Importantly, the manner of the consultation must be non-directive when it identifies TOP as the main option for further management.

### 8.2. Future Directions

It is important to bear in mind that postnatal ventricular drainage is an example of a delayed intervention, even despite reports about relatively favorable developmental outcomes in ISVM children. All changes associated with the destructive effects of VM—anatomopathological, histological, and functional—take place during the prenatal phase of a child’s development. The concept of the 30-year ‘moratorium’ should be revisited due to the advancements in the diagnostic protocols, material bioengineering, and new surgical techniques of prenatal ISVM decompression. The current criteria for SVM isolation seem to be well-defined.

Further studies should focus on the eligibility process of the ISVM candidates for prenatal ventricular decompression, especially how to determine the following: optimal GA, definition of the ventricular volume cut-off point, rate of AD increase, and the thickness of the brain mantle. Ideally, future decompression techniques should offer low surgical risk for the mother and highly effective decompression in the fetus, using a valve system that does not lead to overdrainage. There is a need for a ‘single-step operation’ technique, with a single prenatal implantation of the ventricular valve system and the possibility of repositioning the end of the drain into the peritoneal cavity. ETV appears to be the most promising method as far as the internal drainage systems of CSF are concerned.
